# Transcriptome Profiling Reveals Molecular Responses to Salt Stress in Common Vetch (*Vicia sativa* L.)

**DOI:** 10.3390/plants13050714

**Published:** 2024-03-03

**Authors:** Yanmei Sun, Na Zhao, Hongjian Sun, Shan Xu, Yiwen Lu, Haojie Xi, Zhenfei Guo, Haifan Shi

**Affiliations:** Key Laboratory of State Forestry and Grassland Administration on Grass Germplasm Resources Innovation and Utilization in the Middle and Lower Reaches of the Yangtze River, College of Grassland Science, Nanjing Agricultural University, Nanjing 210095, China; 2019220006@njau.edu.cn (Y.S.); nazhao@njau.edu.cn (N.Z.); 2021120003@stu.njau.edu.cn (H.S.); 2021220001@stu.njau.edu.cn (S.X.); 2021820023@stu.njau.edu.cn (H.X.)

**Keywords:** common vetch, KEGG pathway, lignin biosynthesis, RNA-seq, salt tolerance, soluble sugars

## Abstract

Common vetch (*Vicia sativa* L.) is an important annual diploid leguminous forage. In the present study, transcriptomic profiling in common vetch in response to salt stress was conducted using a salt-tolerant line (460) and a salt-sensitive line (429). The common responses in common vetch and the specific responses associated with salt tolerance in 460 were analyzed. Several KEGG (Kyoto Encyclopedia of Genes and Genomes) pathways, including plant hormone and MAPK (mitogen-activated protein kinase) signaling, galactose metabolism, and phenylpropanoid phenylpropane biosynthesis, were enriched in both lines, though some differentially expressed genes (DEGs) showed distinct expression patterns. The roots in 460 showed higher levels of lignin than in 429. α-linolenic acid metabolism, carotenoid biosynthesis, the photosynthesis-antenna pathway, and starch and sucrose metabolism pathways were specifically enriched in salt-tolerant line 460, with higher levels of accumulated soluble sugars in the leaves. In addition, higher transcript levels of genes involved in ion homeostasis and reactive oxygen species (ROS) scavenging were observed in 460 than in 429 in response to salt stress. The transcriptomic analysis in common vetch in response to salt stress provides useful clues for further investigations on salt tolerance mechanism in the future.

## 1. Introduction

Soil salinity is one of the most important abiotic stresses limiting agricultural development. Salinity inhibits crop growth and development and ultimately reduces crop yields as a result of salt-induced ion toxicity, hyper-osmotic stress, and oxidative damage [1]. The maintenance of intracellular Na^+^ and K^+^ homeostasis, osmolyte accumulation, and the antioxidant defense system are important mechanisms for plant adaptation to salt stress [2]. Soluble sugars are important osmolytes accumulated to stabilize cell membranes for protection against salt-induced osmotic damage [3]. Several enzymes, such as beta-amylase (BAM), beta-glucosidase (BULG), sucrose phosphate synthase (SPS), and trehalose-phosphate synthase (TPS), are involved in salt stress-induced sugar accumulation [4,5,6,7]. The antioxidant defense system is activated through induced expression of the genes encoding superoxide dismutase (SOD), catalase (CAT), and ascorbate peroxidase (APX) under salinity to avoid salt stress-induced ROS accumulation that results in oxidative damage [8]. In addition, lignin deposition in the cell walls that provides structural support and stability to the cells is also associated with salt tolerance. The increased lignification of root cell walls can impede the absorption of external ions by the cells, enhance the structural rigidity and strength of plant conducting tissues, and ultimately improve salt tolerance [9]. 

Common vetch (*Vicia sativa* L.) is an important annual diploid leguminous forage for animal feed due to its high yield, high leaf crude protein, and high digestibility [10]. It is also cultivated as a cover crop in sustainable agricultural systems to decrease the use of fertilizers and reduce CO_2_ emissions due to its capacity to fix atmospheric nitrogen through a symbiotic interaction with rhizobia in soils [11]. Moreover, the seeds of common vetch are considered a new starch and sustainable food source for humans due to their high quantity of starch and crude proteins [12,13]. Agro-morphological traits in common vetch lines, such as forage yield components and morphological variations in seeds, have been evaluated [14,15,16]. A draft genome sequence of common vetch has been reported [17,18], which benefits the identification of important genes. A transcriptomic analysis is performed to reveal the candidate genes involved in pod shattering [19] and responses to Cd, drought, cold, and salt [20,21,22,23,24]. The salt tolerance in 54 lines of common vetch has been evaluated, and the differential physiological responses to salt stress between the salt-tolerant and salt-sensitive lines showed that the capacity for the maintenance of Na^+^ and K^+^ homeostasis, the accumulation of proline, and the activation of the antioxidant defense system is associated with salt tolerance in common vetch [25]. However, the research on the molecular responses in common vetch to abiotic stresses is still limited, and no investigation on molecular responses associated with salt tolerance in common vetch have been conducted.

The objective of this study was to analyze the differentially expressed genes and enriched pathways in response to salinity in common vetch at the transcriptome level using a salt-tolerant and a salt-sensitive line. The results will provide novel information for understanding the molecular responses to salinity in common vetch and identifying important genes conferring salt tolerance for applications to crop improvements.

## 2. Materials and Methods

### 2.1. Plant Materials and Growth

The salt-tolerant line 460 (PI239924) and salt-sensitive line 429 (PI220910) of common vetch were evaluated previously [25] and used in the present study. Uniform seedlings were selected after germination to perform the following hydroponic experiments. The hypocotyls of seedlings were gently wrapped with a sponge and put into a perforated plastic basin filled with 1/2 strength Hoagland nutrient solution (pH 5.8) [26]. The seedlings were growing in a growth chamber at 25 °C and 65% relative humidity with a 16 h/8 h regime under the light of 300 μmol m^−2^ s^−1^, and the nutrient solution was renewed every two days. Eight-day-old seedlings were placed in the nutrient solution containing 150 mM of NaCl for salt treatment, while those growing in the nutrient solution were used as control. 

### 2.2. Measurements of Dry Weight and Leaf Relative Water Content (RWC)

The shoots and roots were harvested and then dried in an oven at 80 °C for 24 h. Their dry weight was then recorded. The leaf RWC was determined according to the standard method proposed by Barrs and Weatherly, 1962 [27]. Fresh leaves were weighed (FW) and in turn, immersed in water overnight to weigh the water-saturated weight (SW), followed by 24 h of drying at 80 °C for weighing the dry weight (DW). The RWC was calculated based on the formula (FW − DW)/(SW − DW) × 100. The experiment was repeated three times.

### 2.3. Measurements of Soluble Sugars

Soluble sugars were determined as described previously [28]. Fresh leaves (0.5 g) were frozen in liquid nitrogen and ground into a powder, followed by the addition of 5 mL of 80% (*v*/*v*) ethanol and incubation in a water bath at 80 °C for 30 min for the extraction of soluble sugars. After cooling to room temperature, the mixture was centrifuged at 12,000× *g* for 20 min. The supernatant was quickly frozen in liquid nitrogen and vacuum-dried and then dissolved in 500 μL of deionized water for filtration through a 0.22 μm Millipore membrane (Millipore, Bedford, MA, USA). Sugars in the filtrate were determined using a Waters 2695 separation system equipped with an amino bonded column (Zorbax NH2; 250 × 4.6 mm; Agilent Technologies Inc., Santa Clara, CA, USA) using a Waters 2414 refractive index detector. Sucrose, glucose, and fructose concentrations were calculated based on the standard curve for each sugar and calibrated according to the recovery throughout the analysis procedure.

### 2.4. Measurement of Total Lignin Content

To obtain a purified cell wall residue, 0.1g leaf or root dry samples were subjected to extractions in 2 mL vials, each time for 30 min, at near-boiling temperatures for water (98 °C), ethanol (76 °C), chloroform (59 °C), and acetone (54 °C). The remaining cell wall residue was dried under a vacuum. The dried cell wall residue was added to a digestion mixture consisting of 2.5 mL of 25% (*w*/*w*) acetyl bromide in glacial acetic acid and 0.1 mL of 70% perchloric acid, and was then heated at 70 °C for 30 min with shaking every 10 min. After cooling on ice, the digested mixture was transferred to a volumetric flask containing 5 mL of 2 M sodium hydroxide and 6 mL of acetic acid and then brought up to a final volume of 25 mL with acetic acid. The acetyl bromide soluble lignin (%ABSL) was quantified by measuring the absorbance at 280 nm. The lignin concentrations were determined using the Bouguer–Lambert–Beer law: A = ε × l × c, with ε = 23.35 l g^−1^ cm^−1^ and l = 0.1 cm.

### 2.5. Lignin Staining

Lignin staining was conducted according to a previous method [29]. The 3 cm root tip samples were cut from seedlings after 3 d of salt treatment and immediately placed in a fixation solution containing 5% formaldehyde, 5% acetic acid, and 50% ethanol for 24 h at room temperature, followed by dehydration in a stepwise manner with 20% ethanol–water mixtures at 60 min intervals until 100% ethanol was reached. Subsequently, the samples were treated with a 30% step-graded series of tert-butyl alcohol (TBA) at 60 min intervals until 100% TBA was reached. The root tips were then infiltrated with paraffin–TBA mixtures and embedded in paraffin for 48 h. Transverse sections of approximately 200 μm thickness were cut from the roots by hand and treated with 95% ethanol for 10 min. Semi-thin sections with a thickness ranging from 5 µm to 8 µm were then prepared by cutting with a microtome blade KD-P (Zhejiang Jinhua Kedi Instrumental Equipment Co., Ltd., Jinhua, China). The lignin was stained using a phloroglucinol hydrochloric acid staining method. The thin paraffin strip sections of root tips were stained by applying an ethanol solution containing 1% phloroglucinol and then subsequently depositing concentrated HCl solution after allowing it to stand for 30 s. The root tip sections were observed using a Nikon ECLIPSE TI-SR microscope (Nikon Instruments, Tokyo, Japan) at 200× magnification.

### 2.6. RNA Isolation, Library Construction, and Sequencing

To avoid potential photoperiod-related effects on gene expression, samples were placed in a growth chamber at 25 °C for 2 h as a control. Total RNA was extracted from 0.1 g of fresh leaves using an RNAprep pure Plant Kit (Tiangen Inc., Beijing, China). After subjecting the plants to salt stress treatment for 2 h and 26 h, a total of 36 samples were collected, consisting of three treatments (0 h, 2 h, and 26 h of salt exposure), two lines (429 and 460), three biological replications, and two different tissues (leaves and roots). A cDNA library was constructed by Gene Denovo Biotechnology Co. (Guangzhou, China) and was sequenced using an Illumina HiSeq TM 2500 (Illumina, San Diego, CA, USA). The RNA-seq data have been deposited in the sequence read archive (SRA) of the NCBI database (accession number: PRJNA1026326). 

### 2.7. Analysis of the Differentially Expressed Genes (DEGs)

After filtering poor-quality reads, clean reads were obtained using a method described previously [21]. The Trinity software (2.6.6) was then used to assemble transcripts. Gene annotation was performed by comparing the sequences with several databases, including NCBI non-redundant protein sequence (NCBI Nr), Clusters of Orthologous Groups of proteins (COG), Swiss Prot, KEGG, and Gene Ontology (GO). DEGs were identified based on a fold change (FC) ≥ 2 and a false discovery rate of *p* < 0.05. GO enrichment and KEGG pathway analyses were performed using the Omicsmart platform (https://www.omicsmart.com/ (accessed on 23 November 2023)).

### 2.8. Quantitative Real-Time PCR (qPCR) Analysis

The RNA sample (1 µg) was reverse-transcribed into first-strand cDNA using the HiScript III RT SuperMix for qPCR Kit (Vazyme, Nanjing, China). The resulting cDNA was diluted and used as a template for qPCR analysis using TSINGKE TSE202 2 × T5 Fast qPCR Mix (TSINGKE, Beijing, China) on a Thermal Cycler DiceTM Real-Time System (Takara, Otsu, Japan). The primers were designed using the online software PrimerQuest Tool (https://sg.idtdna.com/PrimerQuest/Home/Index (accessed on 23 November 2023)) and are listed in Supplementary Table S1. A negative control without a cDNA template was included in every run, and the expression of the common vetch unigene 68614 was used to normalize the amount of template due to its stable expression in common vetch [19]. Relative expression was calculated by 2^−ΔΔCt^ based on two biological replicates, with three technical replicates per biological replicate.

### 2.9. Statistical Analysis

The full randomized design model in the SPSS software (26.0, SPSS Inc., Chicago, IL, USA) was used to analyze the variance of data. Significant differences were calculated based on Duncan’s test at *p* < 0.05.

## 3. Results

### 3.1. Phenotypic Differences between Salt-Tolerant (T) and Salt-Sensitive (S) Strains under Salinity Stress

**Figure 1:**
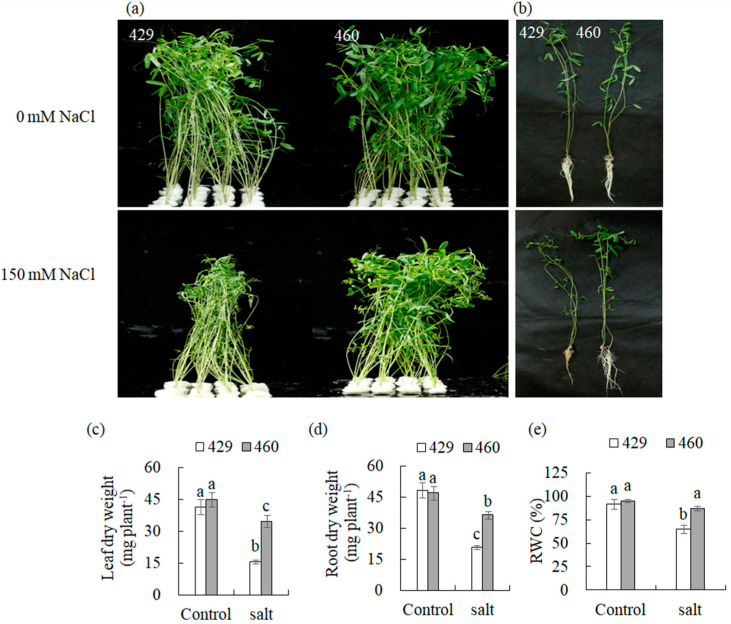
Analysis of salt tolerance in two common vetch lines, 429 and 460. Plant performance was photographed using 8-day-old seedlings of the salt-tolerant line 460 and salt-sensitive line 429 after 3 d of 150 mM NaCl treatment (**a**,**b**), followed by measurements of dry weight of leaves (**c**) and roots (**d**) as well as the relative water content (RWC) in leaves (**e**). Means of fifteen samples and standard errors are presented. The same letter above the columns of each gene indicates no significant difference at *p* < 0.05.

Compared to the unaltered phenotype in the salt-tolerant line (460), leaves in the salt-sensitive line (429) exhibited wilting, yellowing, and shedding when exposed to salt stress (150 mM of NaCl) for 3 d (Figure 1a,b). Salt treatment resulted in a decrease in the dry weight of the leaves and roots in both lines, with less alteration in 460 than in 429. For example, the dry weight of the shoots and roots in 460 was decreased by 22.9% and 21.3%, respectively, while those in 429 were decreased by 62.2% and 56.3%, respectively (Figure 1c,d). The relative water content (RWC) was decreased by 26.9% in 429, while it was not changed in 460 after salt stress treatment (Figure 1e). The results demonstrated the difference in salt tolerance between 460 and 429.

### 3.2. RNA Sequencing, Transcriptome Assembly, and Annotation

To explore the mechanisms underlying the difference in salt tolerance between two lines, a transcriptomic analysis based on deep RNA-seq was performed. After subjecting the plants to salt stress treatment for 2 h and 26 h, total RNA was extracted for further transcriptome sequencing and gene expression profiling analysis. A total of 44,320,236 clean reads were obtained from 44,602,528 raw reads. The clean reads were assembled using the Trinity program to generate a total of 49,712 unigenes after clustering, with 1858 bp of N_50_, a 28.7% GC content and 1158 bp as the average length. The results indicated that the sequencing data and assembly quality were thus appropriate for further analysis.

A total of 30,211 unigenes could be annotated through alignment in four public databases (E-value < 1.0 × 10^−5^), including Nr, Swiss-Prot, KEGG, and KOG. The number of annotated unigenes was arranged from 16,618 (33.4%) in KOG to 29,754 (59.8%) in Nr, while 19,501 (39.2%) unigenes could not be annotated (Figure S1a). The unigenes in common vetch were mostly matched to *Medicago truncatula* (21.7%), followed by *Cicer arietinum* (8.6%) and *Trifolium pratense* (8.4%) (Figure S1b).

### 3.3. DEG Identification and Analysis in Leaves and Roots

**Figure 2:**
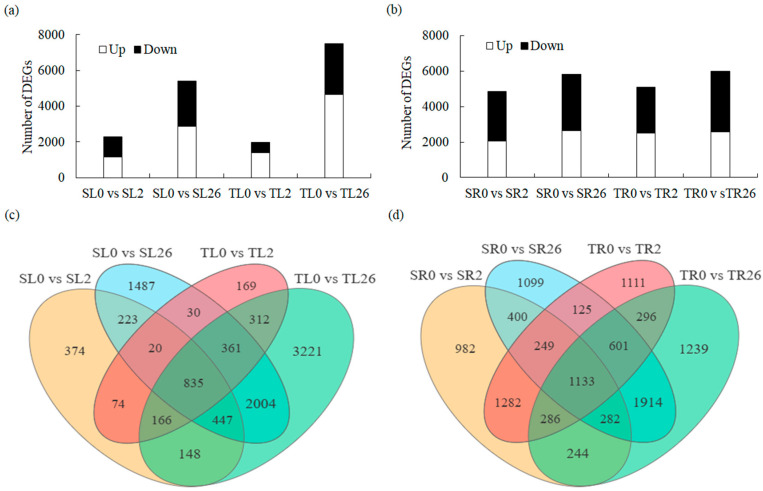
Analysis of the differentially expressed genes (DEGs) in leaves and roots in two common vetch lines in response to salt stress. The number of up-regulated (Up) and down-regulated (Down) DEGs in leaves (**a**) and roots (**b**) in salt-tolerant line 460 and salt-sensitive line 429 after 2 h and 26 h of 150 mM NaCl treatment is summarized. Venn diagrams showed the overlap of DEGs in leaves (**c**) and roots (**d**) of 429 and 460 after 150 mM NaCl treatment for 2 h and 26 h compared with control. Software (https://www.omicshare.com/tools/ (accessed on 23 November, 2023)) was used for the Venn diagram. SL, leaves in salt-sensitive line 429; TL, leaves in salt-tolerant line 460; SR, roots in salt-sensitive line 429; TR, roots salt-tolerant line 460; 0, 1, and 2 represent 0, 2 h, and 26 h after salt treatment, respectively.

A total of 9871 and 11,243 DEGs were identified in the leaves and roots, respectively, based on the screening conditions of FDR < 0.05 and |log2 FC| > 2. After 2 h of salt treatment, 1147 and 1394 DEGs were up-regulated in the leaves of 429 and 460, respectively, whereas 2859 and 4638 DEGs were up-regulated after 26 h of salt treatment in 429 and 460, respectively. On the other hand, the salt treatment resulted in the down-regulation of 1170 DEGs in 429 and 573 DEGs in 460 after 2 h. After 26 h, the number of down-regulated DEGs rose to 2548 in 429 and 2856 in 460 (Figure 2a,c). The results indicated that more genes were up-regulated in the leaves of 460 than in 429 during salt stress, and fewer genes were down-regulated in 460 than in 429 after 2 h of salt stress. In addition, 2062 and 2511 DEGs were up-regulated in the roots of 429 and 460 after 2 h of salt treatment, and 2627 and 2576 DEGs were up-regulated after 26 h of salt treatment, respectively (Figure 2b,d). There were 2796 and 2572 DEGs down-regulated in the roots of 429 and 460 after 2 h of salt treatment. Furthermore, 3176 and 3419 DEGs were down-regulated after 26 h of salt treatment, respectively (Figure 2b,d). The results indicated that more up-regulated and less down-regulated genes were obtained in the roots of 460 than in 429 after 2 h of salt stress.

### 3.4. Analysis of KEGG Pathway Enrichment of DEGs in Response to Salt Stress

**Figure 3:**
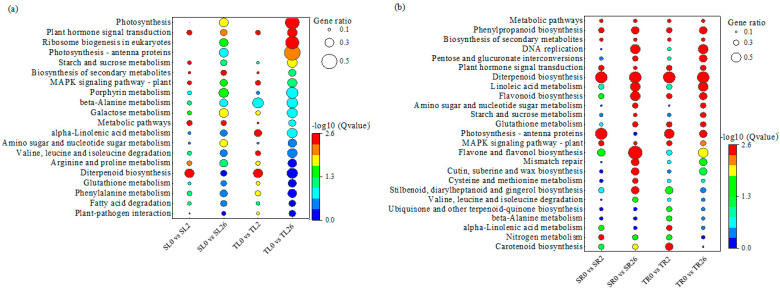
KEGG pathway enrichment analysis of the DEGs in leaves and roots of 429 and 460 in response to salt stress. All the KEGG pathways that are significantly enriched in leaves (**a**) and roots (**b**) in salt-tolerant line 460 and salt-sensitive line 429 after 2 h and 26 h of salt treatment compared with 0 h are presented. Y-axis represents KEGG enrichment terms. The color of the dot represents −log_10_ (Q value). It was shown that the statistical significance increased from blue to red (red represents high significance, while blue represents low). The size of the dot represents the ratio of enriched DEGs to background genes. SL, leaves in salt-sensitive line 429; TL, leaves in salt-tolerant line 460; SR, roots in salt-sensitive line 429; TR, roots salt-tolerant line 460; 0, 1, and 2 represent 0, 2 h, and 26 h after salt treatment, respectively.

KEGG enrichment analysis was performed to understand the gene networks associated with the differential salt tolerance between two lines. The DEGs in the leaves of both 460 and 429 after 2 h of salt stress were enriched in six KEGG pathways, including plant hormone signal transduction, the MAPK signaling pathway, the biosynthesis of secondary metabolites, terpenoid biosynthesis, galactose metabolism, and arginine and proline metabolism (Figure 3a). Plant hormone signal transduction, photosynthesis, and eukaryotic ribosomes were enriched in the leaves of both 460 and 429 after 26 h of salt treatment. The DEGs in the above pathways reflected the common responses to salinity in the leaves in both lines (Figure 3a). Five KEGG pathways, including alpha-linolenic acid metabolism; valine, leucine, and isoleucine degradation; plant–pathogen interaction; glutathione metabolism; and phenylalanine metabolism, were specifically enriched in 460 after 2 h of salt stress, while the starch and sucrose metabolism pathway was specifically enriched in 429 after 2 h of salt stress (Figure 3a). The photosynthesis-antenna and starch and sucrose metabolism pathways were specifically enriched in 460 after 26 h of salt stress, while metabolic pathways, amino sugar and nucleotide sugar metabolism, secondary metabolite biosynthesis, galactose metabolism, and the MAPK signaling pathway were specifically enriched in 429 after 2 h of salt stress (Figure 3a). The difference in the above pathways between the two lines reflect the specific responses to salinity in the leaves associated with the differential salt tolerance lines.

A total of ten KEGG pathways were enriched in roots of both 460 and 429 after 2 h of salt stress, including metabolic pathways, secondary metabolite biosynthesis, plant hormone signal transduction, phenylpropanoid biosynthesis, the MAPK signaling pathway, terpenoid biosynthesis, alpha-linolenic acid metabolism, flavonoid biosynthesis, zeatin biosynthesis, and nitrogen metabolism (Figure 3b). After 26 h of salt stress, sixteen KEGG pathways were significantly enriched in the roots of both 460 and 429, including metabolic pathways, phenylpropanoid biosynthesis, secondary metabolite biosynthesis, DNA replication, the interconversion of pentose and glucuronate, plant hormone signal transduction, terpenoid biosynthesis, linoleic acid metabolism, flavonoid biosynthesis, amino sugar and nucleotide sugar metabolism, starch and sucrose metabolism, glutathione metabolism, photosynthesis-antenna proteins, the MAPK signaling pathway, flavonoid and flavonol biosynthesis, and mismatch repair (Figure 3b). The DEGs in the above pathways reflect the common responses to salinity in the roots in both lines.

Seven pathways, including carotenoid biosynthesis; glutathione metabolism; nitrogen metabolism; stilbenoid, diarylheptanoid, and gingerol biosynthesis; ubiquinone and other terpenoid-quinone biosynthesis; and beta-alanine metabolism, were specifically enriched in the roots of 460 after 2 h of salt stress, while flavonoid and flavonol biosynthesis-related pathways were specifically enriched in the roots of 429 after 2 h of salt stress (Figure 3b). No pathway was specifically enriched in the roots of 460 after 26 h of salt stress, while six pathways, including carotenoid biosynthesis; nitrogen metabolism; the degradation of valine, leucine, and isoleucine; the biosynthesis of stilbenoids, diarylheptanoids, and gingerols; cysteine and methionine metabolism; and the biosynthesis of cutin, suberin, and wax, were specifically enriched in the roots of 429 (Figure 3b). The difference in the above pathways between the two lines reflect the specific responses to salinity in roots associated with the differential salt tolerance.

### 3.5. Plant Hormone and MAPK Signaling Pathways Are Common for Common Vetch in Response to Salt Stress

The plant hormone signaling transduction pathway in common vetch in response to salt was further analyzed. The ABA and IAA signaling transduction pathways were enriched in both lines after salt stress. Among the members in ABA signaling, bZIP transcription factors *TRAB1/ABF2* and *SRK2A* and abscisic acid receptor genes *PYL3* and *PYL4* were up-regulated in the leaves of both 429 and 460, with higher levels in 460 than in 429 (Figure S2a). *PYL1* was down-regulated in the roots of both 429 and 460 due to salinity stress after 2 and 26 h, but they were induced at higher levels in 429 than in 460. *ABF2* and the serine/threonine protein kinase *SAPK8*, *SRK2E*, and *PYL4* genes were up-regulated in roots after 2 h and 26 h of salt stress, with higher levels in 460 than in 429. *SAPK10* and *PYL1* were down-regulated in the roots of both 429 and 460 due to salinity stress after 2 and 26 h, but they were induced at higher levels in 429 than 460 (Figure S2b). *PYL8* was down-regulated in the leaves and roots of both 429 and 460 due to salinity stress after 2 and 26 h, but they were induced at higher levels in 429 than 460 (Figure S2a,b).

Among the members involved in IAA signaling, the auxin response factors *IAA1*, *IAA12*, *IAA19*, *IAA26*, *ARF3*, and *ARF9* were up-regulated in the leaves of both 429 and 460 after 2 h and 26 h of salt stress, with higher levels in 460 than in 429. *IAA30*, *SAUR20*, and *SAUR50* were down-regulated in the leaves of both 429 and 460 due to salinity stress after 2 and 26 h, but they were induced at higher levels in 429 than 460 (Figure S2a). *SAUR40*, *SAUR71*, *IAA1*, and *IAA26* were up-regulated in the roots of both 429 and 460 after 2 h and 26 h of salt stress, with higher levels in 460 than in 429. *IAA6*, *IAA9*, *IAA14*, *SAUR20*, and *SAUR50* were down-regulated in the roots of both 429 and 460 due to salinity stress after 2 and 26 h, but they were induced at higher levels in 429 than 460 (Figure S2a,b).

The mitogen-activated protein kinase genes *MKK9*, *MAPKKK17*, and *MEKK1* were up-regulated in the leaves of both 429 and 460 after 2 and 26 h of salt treatment, with higher levels in 460 than in 429 (Figure S2c). The transcript levels of *MMK2* and *MPK3* were down-regulated by salinity stress after 2 h, but they were induced at higher levels in 429 than 460. *MMK2* and *MPK3* were up-regulated after 26 h in both 429 and 460, with higher levels in 460 than in 429 (Figure S2c). In the roots, the *MEKK1* and *MKK1* genes were up-regulated in both 429 and 460 after 2 and 26 h of salt treatment, with higher levels in 460 than in 429 (Figure S2d). The *MPK3*, *MKK9*, and *MKS1* genes were down-regulated in both 429 and 460 after 2 and 26 h of salt treatment, with lower levels in 429 than in 460. The transcript levels of *MAPKKK17* were up-regulated after 2 h in both 429 and 460, with higher levels in 460 than in 429; *MAPKKK17* was down-regulated by salinity stress after 2 h, but it were induced at higher levels in 429 than 460 (Figure S2d). 

### 3.6. Phenylpropanoid Biosynthesis Is Involved in Response to Salt Stress in Common Vetch

The phenylpropanoid biosynthesis in common vetch in response to salt stress was further analyzed. The number of DEGs related to phenylpropanoid biosynthesis in the roots was significantly higher than in the leaves, and the number of up-regulated genes in the roots of 460 was significantly higher than in 429 (Figure S3). *Phenylalanine ammonialyase 1* (*PAL1*), *4-coumarate:CoA ligase* (*4CL*), *4-coumarate:CoA ligase* 7 (*4CLL7*), *cinnamyl alcohol dehydrogenase 1* (*CAD1*), *peroxidases PER17*, *PER43*, *PER52*, *PER52#1*, *PER72*, and *p-hydroxycinnamoyl-CoA:quinate/shikimate p-hydroxycinnamoyl transferase* (*HST*) were up-regulated in both varieties after 2 and 26 h of salt treatment, with higher levels in 460 than in 429 (Figure S3a). *Spermidine hydroxycinnamoyl transferase* (*SHT)* was significantly up-regulated in the leaves of 460 but down-regulated in 429 after 2 and 26 h of salt stress (Figure S3a). *PAL1*, *PAL2*, *PAL17.1*, *4CLL1*, *cinnamoyl-CoA reductase 1* (*CCR1*), *cinnamyl alcohol dehydrogenase 2* (*CCR2*), *CAD1*, *CAD2*, *PER52#1*, *PER54*, *PER65*, *CYP84A1*, *caffeic acid O-methyltransferase* (*COMT*), *caffeoyl-CoA O-methyltransferase* (*CCOMT#1*), *HST*, and *omega-hydroxypalmitate O-feruloyl transferase* (*HHT1*) were up-regulated in the roots of both lines after 2 and 26 h of salt treatment, with higher levels in 460 than in 429. *4CL7*, *4CLL1*, *PER42*, *PER47*, *CCOMT*, and *caffeoyl-CoA O-methyltransferase 2* (*CCOAOMT2*) were up-regulated in 460 but down-regulated in 429 after 2 and 26 h of salt treatment (Figure S3b). 

**Figure 4:**
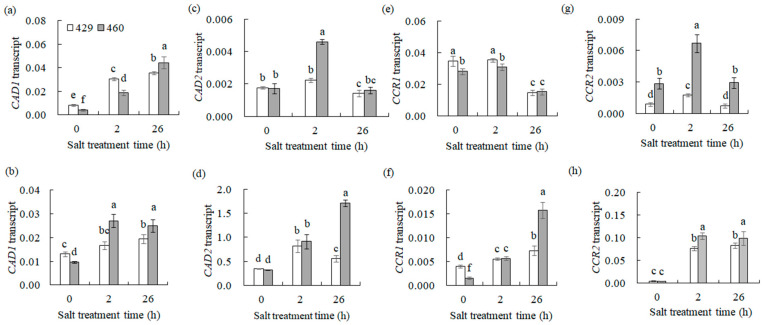
Transcript levels of the genes involved in lignin synthesis in two common vetch lines in response to salt stress. The transcription level of *CAD1* (**a**,**b**), *CAD2* (**c**,**d**), *CCR1* (**e**,**f**), and *CCR2* (**g**,**h**) was detected in salt-tolerant line 460 and salt-sensitive line 429 after 0 h, 2 h, and 26 h of NaCl treatment using qRT-PCR analysis. The data from leaves are presented in (**a**,**c**,**e**,**g**), while those from roots are presented in (**b**,**d**,**f**,**h**). Means of three independent samples and standard errors are presented. The same letter above the columns of each gene indicates no significant difference at *p* < 0.05.

**Figure 5:**
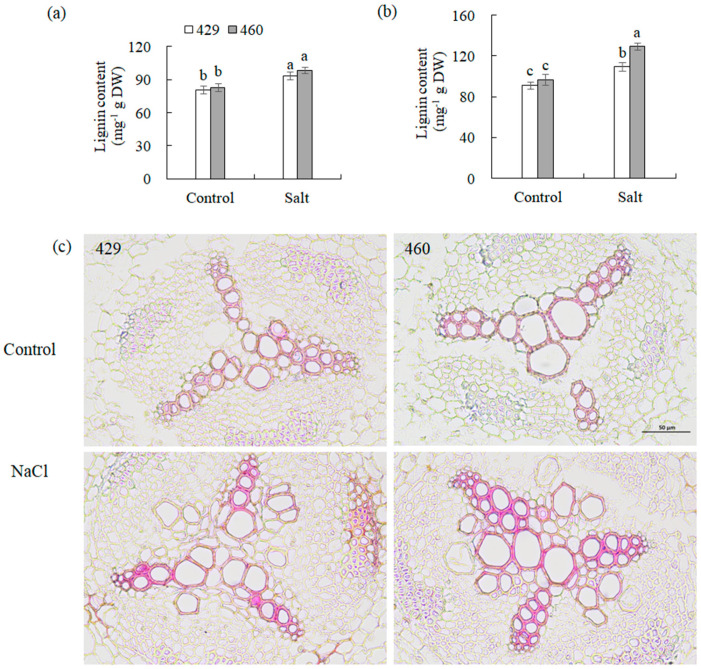
Analysis of lignin in two common vetch lines in response to salt stress. Lignin content in leaves (**a**) and roots (**b**) of salt-tolerant line (460) and salt-sensitive line (429) was measured after 3 d of 150 mM NaCl treatment. Histochemical analysis of lignin in root cross sections was conducted using phloroglucinol (**c**). Three independent experiments were carried out, and similar results were obtained (**c**). Means of three independent samples and standard errors are presented. The same letter above the columns of each gene indicates no significant difference at *p* < 0.05. Scale bar in (**c**) = 50 µm.

Given that *CCR1*, *CCR2*, *CAD1*, and *CAD2* are key enzymes involved in lignin biosynthesis, their gene expression alteration should affect lignin biosynthesis. The transcript levels of these lignin synthesis-related genes and lignin content in 429 and 460 when affected by salt stress were analyzed. *CAD1* was up-regulated, and *CAD2*, *CCR1*, and *CCR2* were down-regulated in the leaves of both lines after 26 h of salt treatment (Figure 4a,c,e,g). *CCR1*, *CCR2*, *CAD1*, and *CAD2* were up-regulated in the roots of both 460 and 429 after 26 h of salt treatment (Figure 4b,d,f,h). The lignin content showed no significant difference in the leaves and roots between the two lines under the control condition. It was increased in the both leaves and roots of the two lines after 3 d of salt treatment, with a higher level in the roots of 460 than in 429 but no difference in the leaves of the two lines (Figure 5a,b). The changes in lignin content were consistent with the altered gene expression (Figure 4). The lignin staining using phloroglucinol showed a similar level of lignin in 460 and 429 under the control condition, while a higher level of lignin was observed in 460 than in 429 after 3 d of salt stress (Figure 5c).

### 3.7. Analysis of Salt-Responsive DEGs Associated with the Higher Salt Tolerance in 460 Than in 429

#### 3.7.1. Alpha-Linolenic Acid Metabolism

After 2 and 26 h of salt treatment, the expression level of the genes involved in alpha-linolenic acid metabolism, including *allene oxide cyclase* (*AOC*); *allene oxide synthase1* (*AOS1)*; *α-dioxygenase 1* (*DOX1)*; *peroxisomal fatty acid beta-oxidation multifunctional protein AIM1*; *acyl-coenzyme A oxidase ACX2*, *ACX3*, and *ACX4*; *linoleate 13S-lipoxygenase LOX2.1*; *fatty acid β-oxidation multifunctional protein 2* (*MFP2*); *12-oxophytodienoic acid reductases OPR1*, *OPR3*, and *OPR11*; and *SDP1,* were up-regulated in the leaves of both lines, with higher levels in 460 than in 429 (Figure S4a). *ACX3*, *ACX4*, *AOC*, *AOS1*, *LOX2.1*, *LOX3.1*, *MFP2*, *OPR1*, *OPR3*, and *OPR11* were up-regulated in the roots of both 429 and 460 after 2 and 26 h of salt treatment, with higher levels in 460 than in 429 (Figure S4b). The results indicated that alpha-linolenic acid metabolism was probably associated with higher salt tolerance in 460 than in 429.

#### 3.7.2. Photosynthesis-Antenna Pathway

The genes encoding the leaf chlorophyll binding proteins LHCB1.3, LHCB3, LHCB2.1, LHCA3, and LHCB7 in the photosynthesis-antenna pathway were significantly up-regulated in the leaves of 460, but down-regulated in 429 after 2 and 26 h of salt stress (Figure S4c). *LHCA1*, *LHCA2*, *LHCA4*, *LHCB1.1*, *LHCB1.3*, *LHCB2.1*, *LHCB3*, *LHCB4.1*, *LHCB4.2*, and *CAP10B* were significantly up-regulated in the roots of 460 but down-regulated in 429 after 2 and 26 h of salt stress (Figure S4d).

#### 3.7.3. Carotenoid Biosynthesis

*9-cis-epoxycarotenoid dioxygenase 1* (*NCED1*), *zeaxanthin epoxidase* (*ZEP*), and *cytochrome P450 hydroxylase* (*CYP707A1*) in the carotenoid biosynthesis pathway were up-regulated in the leaves of both lines after 2 and 26 h of salt treatment, with higher levels in 460 than in 429 (Figure S4e). *NCED1*, *NCED2*, *phytoene synthases PSY* and *PSY1*, *CYP707A2*, and *lutein epsilon hydroxylase 2* (*LUT2*) were up-regulated in the roots of both lines after 2 h of salt treatment, with higher levels in 460 than in 429 (Figure S4f).

#### 3.7.4. Starch and Sucrose Metabolism

**Figure 6:**
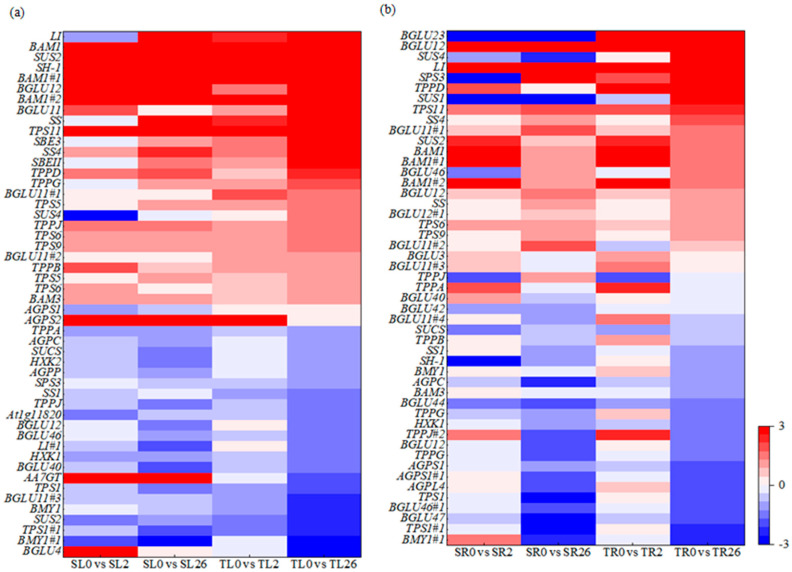
Heatmap of the DEGs involved in starch and sucrose metabolism in two common vetch lines in response to salt stress. The DEGs involved in starch and sucrose metabolism in leaves (**a**) and roots (**b**) were presented. The color scale represents log_2_-transformed FPKM (fragments per kilobyte per million reads) values. The gradual change in color indicates the different expression levels of DEGs. Red indicates up-regulation, and blue indicates down-regulation. SL, leaves in salt-sensitive line 429; TL, leaves in salt-tolerant line 460; SR, roots in salt-sensitive line 429; TR, roots salt-tolerant line 460; 0, 1, and 2 represent 0, 2 h, and 26 h after salt treatment, respectively.

The DEGs in starch and sucrose metabolism were further analyzed. *Sucrose synthase 4* (*SUS4*), *sucrose synthase 2-like* (*SUS2*), *sucrose synthase isoform 3* (*SUS3*), Sucrose synthase 1 (SH-1), *beta-glucosidase 11* (*BGLU11*), *beta-glucosidase* (*LI*), *starch synthase 4* (*SS4*), *trehalose-phosphate synthase 9* (*TPS9*), *trehalose-phosphate synthase 6* (*TPS6*), *trehalose-phosphate synthase 5* (*TPS5*), *trehalose-phosphate synthase 11* (*TPS11*), *trehalose-phosphate synthase G* (*TPPG*), *trehalose-phosphate synthase J* (*TPPJ*), *trehalose-phosphate synthase D* (*TPPD*), *beta-amylase 1* (*BAM1*), and *starch branching proteins SBEII* and *SBE3* were up-regulated in the leaves after 2 h and 26 h of salt stress, with higher expression levels in 460 than in 429 (Figure 6a). *Sucrose-UDP glucosyltransferase* (*SUCS*), *TPPA*, and *beta-amylase1* (*BMY1*) were down-regulated in the leaves after 2 h and 26 h of salt stress, with lower levels in 460 than in 429 (Figure 6a). The genes *SUS4*, *SUS1*, SS4, *SUS2*, *SS*, *BGLU3*, *LI*, *BGLU11#1*, *BGLU11#2*, *BGLU12*, *BGLU12#1*, *BAM1*, *BAM1#1*, *BAM1#2*, *sucrose-phosphate synthase* (*SPS3*), *TPPD*, *TPS11*, and *TPS6* were up-regulated in the roots of both lines after 2 and 26 h of salt treatment, with higher levels in 460 than in 429 (Figure 6b).

**Figure 7:**
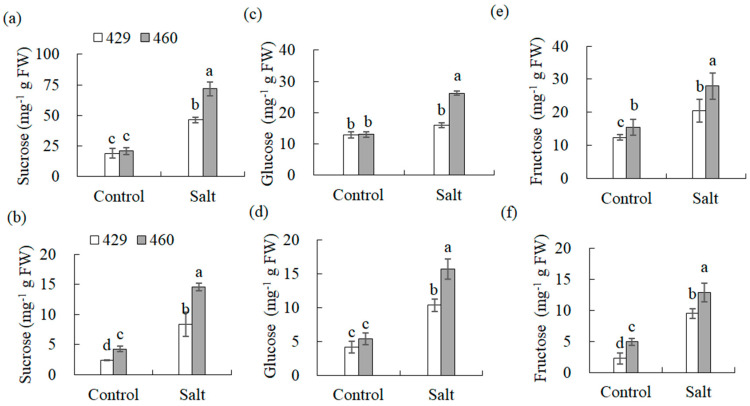
Analysis of soluble sugars in two common vetch lines in response to salt stress. Sucrose (**a**,**b**), glucose (**c**,**d**), and fructose (**e**,**f**) in salt-tolerant line 460 and salt-sensitive line 429 were measured after 3 d of 150 mM NaCl treatment. The data from leaves are presented in (**a**,**c**,**e**), while those from roots are in (**b**,**d**,**f**). Means of three independent samples and standard errors are presented. The same letter above the columns of each gene indicates no significant difference at *p* < 0.05.

Soluble sugars in the leaves and roots of 429 and 460 were analyzed. There was no significant difference in the sucrose and glucose levels in the leaves and the glucose levels in the roots between lines 460 and 429 under the control condition (Figure 7a,c,d), and slightly higher levels of fructose in the leaves and sucrose and fructose in the roots were observed in line 460 than in 429 (Figure 7b,e,f). The levels of soluble sugars were all increased in the leaves and roots of both lines after salt stress, with higher levels in 460 than in 429 (Figure 7a–f).

#### 3.7.5. Na^+^ and K^+^ Transport and ROS Scavenging

**Figure 8:**
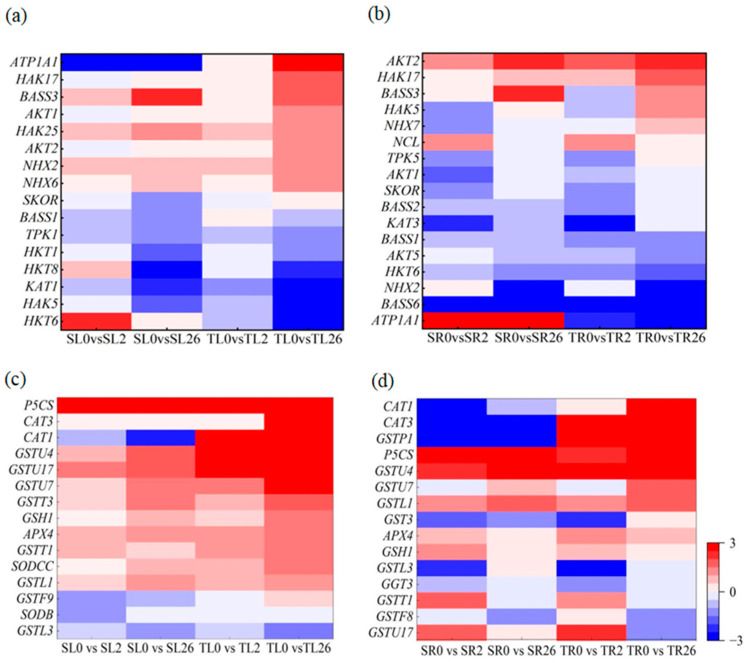
Heatmap of DEGs involved in Na^+^ and K^+^ transport and ROS scavenging in two common vetch lines in response to salt stress. The DEGs involved in Na^+^ and K^+^ transport in leaves (**a**) and roots (**b**) and ROS scavenging in leaves (**c**) and roots (**d**) are presented. The color scale represents log_2_-transformed FPKM (fragments per kilobyte per million reads) values. The gradual change in color indicates the different expression levels of DEGs. Red indicates up-regulation, and blue indicates down-regulation. SL, leaves in salt-sensitive line 429; TL, leaves in salt-tolerant line 460; SR, roots in salt-sensitive line 429; TR, roots in salt-tolerant line 460; 0, 1, and 2 represent 0, 2 h, and 26 h after salt treatment, respectively.

Given that Na^+^ and K^+^ homeostasis, proline accumulation, and antioxidant defense are associated with higher salt tolerance in 460 than in 429 [25], DEGs involved in Na^+^ and K^+^ transport, the glutathione metabolism pathway, antioxidant enzymes, and proline biosynthesis were analyzed. Among the DEGs involved in Na^+^ and K^+^ transport, *HKT1*, *HAK17*, *AKT1*, *HAK25*, *AKT2*, *NHX2*, *NHX6*, and *SKOR* were up-regulated in the leaves after 2 and 26 h of salt stress, with higher levels in 460 than in 429 (Figure 8a). *NHX7*, *AKT2*, *HAK17*, *HAK5*, and *TPK5* were up-regulated in the roots after 2 and 26 h of salt stress, with higher levels in 460 than in 429. *AKT1*, *KAT3*, *HKT6*, and *NHX2* were down-regulated in the roots after 2 h and 26 h of salt stress, with lower levels in 460 than in 429 (Figure 8b). 

The *Glutathione S-transferase* genes *GSTU4*, *GSTU7*, *GSTU17*, *GSTT1*, *GSTT3,* and *GSTL1* and *glutamylcysteine synthetase1* (*GSH1*) were up-regulated in the leaves of both lines after 2 h and 26 h of salt stress, with higher levels in 460 than in 429 (Figure 8c). *GST3* and *GSTP1* were up-regulated in the roots of 460 but down-regulated in 429 after 2 and 26 h of salt stress. *GSTU4* and *GSTL1* were up-regulated in the roots of both lines after 26 h of salt treatment, with higher levels in 460 than in 429 (Figure 8d). *CAT3* and *APX4* were up-regulated in the leaves of both lines after 2 and 26 h of salt treatment, with higher levels in 460 than in 429. *CAT1* was up-regulated in the leaves of 460 but down-regulated in 429 after 2 and 26 h of salt stress. *SODCC* was down-regulated in the leaves after 2 h and 26 h of salt stress, with lower levels in 460 than in 429 (Figure 8c). *CAT1* and *CAT3* were up-regulated in the roots of 460 but down-regulated in the roots of 429 after 2 and 26 h of salt treatment. *APX4* was up-regulated in the roots of both lines after 2 and 26 h of salt treatment, with higher levels in 460 than in 429 (Figure 8d). *P5CS* was up-regulated in the leaves and roots of both lines after 2 and 26 h of salt treatment, with higher levels in 460 than in 429 (Figure 8c,d).

**Figure 9:**
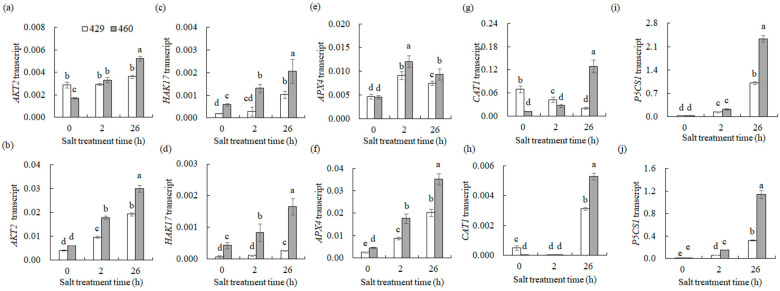
Transcript levels of genes involved in ion homeostasis and ROS scavenging in two common vetch lines in response to salt stress. The transcription levels of *AKT2* (**a**,**b**), *HAK17* (**c**,**d**), *APX4* (**e**,**f**), *CAT1* (**g**,**h**), and *P5CS1* (**i**,**j**) were detected in salt-tolerant line (460) and salt-sensitive line (429) after 0, 2, and 26 h of NaCl treatment using qRT-PCR analysis. The data from leaves are presented in (**a**,**c**,**e**,**g**,**i**), while those from roots are in (**b**,**d**,**f**,**h**). Means of three independent samples and standard errors are presented. The same letter above the columns of each gene indicates no significant difference at *p* < 0.05.

Some of the above genes were verified using qPCR (Figure 9). Under control conditions in the leaves, the expression of *AKT2* and *CAT1* in 460 was lower than that in 429 (Figure 9a,g). However, *HAK17* expression in 460 was higher than that in 429 (Figure 9c), while *APX4* and *PSCS1* expression displayed no difference between 429 and 460 (Figure 9e,i). After 2 h of salt treatment, *AKT2* expression was up-regulated only in 460 but the gene expression showed no difference between 460 and 429. *HAK17* and *APX4* expression were increased in both lines following 2 h of salt treatment, with higher levels observed in 460 than in 429 (Figure 9c,e). After 26 h of salt treatment in the leaves, *AKT2* expression was up-regulated only in 460. *HAK17*, *APX4*, and *P5CS1* expression in leaves increased in both lines, with higher levels in 460 than in 429 (Figure 9a,c,i). *CAT1* expression was reduced in 429 but increased in 460, resulting in a higher expression level in 460 than in 429 (Figure 9g). Under control conditions in the roots, no difference was observed in *AKT2* and *P5CS1* expression between 429 and 460 (Figure 9a,j). However, *HAK17* and *APX4* expression in 460 were higher than that in 429 (Figure 9c), while *CAT1* expression was lower in 460 than in 429 (Figure 9h). After 2 h of salt treatment in roots, the expression of *AKT2*, *APX4*, and *P5CS1* was up-regulated in both lines, with higher levels in 460 than in 429 (Figure 9b,f,j). *HAK17* expression was increased only in 460, resulting in a higher level in 460 (Figure 9d). After 26 h of salt treatment in roots, the expression of *AKT2*, *HAK17*, *APX4*, *CAT1*, and *P5CS1* were increased in both lines, with higher levels in 460 than in 429 (Figure 9b,d,f,h,j). The results here are consistent with the transcriptome data above, suggesting that the distinct expression patterns of genes involved in Na^+^ and K^+^ transport and ROS scavenging might contribute to the salt tolerance between the two lines.

## 4. Discussion

### 4.1. Plant Hormone and MAPK Signaling Were Associated with Salt Tolerance in Common Vetch

ABA is a central integrator to activate adaptive signaling cascades during the salt stress response in plants [30]. The ABA signaling pathway involves the recognition of ABA by PYRABACTIN RESISTANCE/PYRABACTIN RESISTANCELIKE (PYR/PYL) receptors, the inactivation of PP2C/ABI1, and the subsequent activation of subclass III SnRKs. The overexpression of ABA receptor genes leads to enhanced salt tolerance. For example, overexpression of rice *OsPYL5* and grape *VaPYL4* enhances salt tolerance in Arabidopsis, respectively [31,32]. Various transcription factors, such as ABF, act downstream of SnRK2 to regulate the synthesis of compatible osmolytes in response to salt stress [33,34]. As a growth-promoting hormone, IAA regulates root developmental plasticity, which is considered a key adaptive trait in the plant response to abiotic stresses [35,36]. Salt-inhibited root growth is associated with reduced IAA accumulation, which may be attributed to the polar transport of IAA [37,38]. *AUX/IAA* genes play a central role in auxin signal transduction by acting as early auxin response genes that modulate the expression of auxin response genes through interactions with ARFs. AUX/IAA genes are involved in the salt tolerance of plants. The heterologous overexpression of *OsIAA18* enhances salt tolerance and osmotic stresses in transgenic Arabidopsis plants [39]. *OsIAA20* plays a positive role in salt stress responses in an ABA-dependent manner [40]. The ABA and IAA signaling transduction pathways were enriched in both common vetch lines in the present study, suggesting the involvement of ABA and IAA signaling in the adaptive response of common vetch to salt conditions. Distinct expression patterns of many DEGs involved in IAA and ABA signaling, such as PYLs, IAAs, SnRKs, and ARFs, were observed in the two lines, suggesting that the ABA and IAA signaling pathways are likely associated with the differential salt tolerance between the two lines. These results are similar to those reported for maize [41], which showed that ABA and IAA signaling pathway-associated genes were enriched and presumed to be involved in the salt response.

The MAPK cascade is a conserved signaling pathway involved in the regulation of the salt stress signaling pathway [42]. The integration of MAPK signal transduction with the biosynthesis, transport, and signal transduction of plant hormones plays a critical role in regulating stress responses and coordinating plant growth and development [43]. The overexpression of *OsMKK6* enhances salt tolerance in rice with enhanced root and shoot lengths and weights and MBP phosphorylation activity under salt stress [44]. The *AtMKK9-AtMPK3*/*AtMPK6* cascade participates in the regulation of ethylene and camalexin biosynthesis in Arabidopsis [45]. MPK6 phosphorylates the Na^+^/H^+^ antiporter protein SOS1 [46]. Overexpression of *VvMAPK9* significantly enhances salt tolerance in *Arabidopsis* [47]. The *MAPKKK*, *MKK*, and *MPK* genes displayed distinct expression profiles in both the leaves and roots in the two lines, with higher expression levels of *MKK9*, *MAPKKK17*, and *MEKK1* in 460 than 426 after 2 h and 26 h of salt treatment. The results suggest that the MAPK pathway is associated with the differential salt tolerance between the two lines.

### 4.2. Phenylpropanoid Biosynthesis Was Associated with Salt Tolerance in Common Vetch

Lignin is synthesized from phenylalanine through the phenylpropanoid pathway, including the reactions catalyzed by phenylalanine ammonia-lyase (PAL), cinnamate 4-hydroxylase (C4H), 4-coumarine-CoA ligase (4CL), cinnamyl alcohol dehydrogenase (CAD), peroxidase (POX), and laccase (LAC) [48,49]. PAL, C4H, 4CL, and CAD are major rate-limiting enzymes in lignin biosynthesis, and they are involved in salt tolerance [50,51]. Multiple genes involved in lignin synthesis showed up-regulation in the roots when subjected to salt treatment in common vetch, and higher expression levels were shown in 460 than in 429. Consistently, the lignin content was higher in the roots of 460 than in 426. Salt stress triggers the phenylpropanoid biosynthesis pathway, leading to increased cell lignification [52]. *4CL*, *CAD*, *CCR*, *COMT*, and CCoAOMT expression and lignin monomer p-coumaryl alcohol and sinapyl alcohol levels in *Sesuvium portulacastrum* were increased, which leads to accumulated lignin in roots under salt stress [53]. This lignification process obstructs the uptake of external ions by root cells, thereby enhancing the structural rigidity and strength of plant conducting tissues for improving salt tolerance [9]. Thus, the increased lignin content in the roots was associated with the higher salt tolerance in 460 than in 429. Interestingly, lignin accumulation in the roots of salt-tolerant Chinese cabbage was also found to be an important strategy for resisting salt stress [54].

### 4.3. The Carbohydrate and Energy Metabolisms Were Associated with the Differential Salt Tolerance between Two Lines

A considerable number of differentially expressed genes (DEGs) participating in various carbohydrate and energy metabolism pathways were observed in variety 460 in response to salinity, including starch and sucrose metabolism, alpha-linolenic acid metabolism, carotenoid biosynthesis, and photosynthesis-antenna proteins. Sugars in plants act as osmolytes to alleviate the negative effects of salt stress [55]. In this study, a series of genes involved in starch and sucrose metabolism were up-regulated after salt treatment. Sucrose synthase (SUS) is a key enzyme in sucrose metabolism in higher plants. β-amylase (BAM) catalyzes the conversion of starch into soluble sugars [6]. β-glucosidase (BGLU) is a rate-limiting enzyme involved in cellulose hydrolysis, breaking down cellulose into glucose units [5]. Transcriptomic analysis of oats has shown that genes encoding β-glucosidase-related enzymes are significantly up-regulated under salt stress in salt-tolerant varieties. The *SUS4*, *SUS2*, *SS*, *SH-1*, *BAM*, *BGLU11*, and *LI* expression levels were higher in 460 than in 429 under salt stress, which was associated with higher levels of sucrose, fructose, and glucose levels in 460 than in 429. The difference in gene expression levels and soluble sugar accumulation may be associated with the differential salt tolerance in 460 and 429. Furthermore, genes related to trehalose synthesis (*TPS9*, *TPS6*, *TPS5*, *TPS11*, *TPPG*, *TPPJ*, *TPPD*) were up-regulated in response to salt stress, with higher transcript levels in 460. Trehalose forms a unique protective structure on the cell membrane surface, which preserves the activity of functional protein molecules and enables resistance to various abiotic stresses [7]. The results here are similar to those reported in Halophytic *Eutrema salsugineum*, in which sucrose, trehalose, raffinose, and xylose, as well as the transcript levels of related DEGs, were significantly enhanced [56].

It has been well-documented that the regulation of alpha-linolenic acid metabolism and carotenoid biosynthesis plays a crucial role in salt tolerance in plants. Alpha-linolenic acid is a major unsaturated fatty acid in plants [57]. Polyunsaturated fatty acids play a key role in maintaining the structure and function of the cell membrane, contributing to their ability to respond and adapt to changing environmental conditions. Unsaturated fatty acids are associated with salt tolerance in *Suaeda salsa* and tomato [58,59]. Carotenoids play essential roles in photosystem assembly, light harvesting, and photoprotection [60]. The increased carotenoid contents lead to improved abiotic stress tolerance by scavenging ROS [61]. The photosynthetic antenna complex plays a crucial role in influencing downstream energy-related processes, thereby exerting a profound influence on overall plant growth and development [62]. Plants have developed diverse strategies for tolerance to salt stress, but the maintenance of an adequate energy supply is a prerequisite for their survival under salt stress conditions [63]. In the present study, we observed that genes related to the photosynthetic antenna complex were up-regulated following 26 h of salt treatment, while significant down-regulation was observed in the line 429. This observed difference suggests that salt stress has different impacts on the photosynthetic antenna complex in the 460 and 429 lines. A comparative proteomic analysis of two sesame genotypes with contrasting salinity tolerance showed that carbon fixation in photosynthetic organisms, carbon metabolism, alpha-linolenic acid metabolism, and photosynthesis were involved in the salt response [64], which were similar to those results here. Consequently, the line 460 can maintain its photosynthetic ability and ensure an uninterrupted energy supply, ultimately leading to improved salt tolerance.

### 4.4. Na^+^ and K^+^ Transport and ROS Scavenging Were Associated with the Differential Salt Tolerance between the Two Lines

Salinity leads to the accumulation of Na^+^ and ionic toxicity in common vetch [22,65]. The intracellular Na^+^ and K^+^ ion balance plays a crucial role in plant salt tolerance. Na^+^ and K^+^ channels and transporters are key players in the maintenance of Na^+^ and K^+^ homeostasis [2]. *AKT2*, *HAK17*, *AKT1*, and *NHX2* were up-regulated in the leaves and roots of both 460 and 429 in response to salinity. *HKT1*, *HAK25*, *NHX6*, and *SKOR* were specifically up-regulated in the leaves, whereas *NHX7*, *HAK5*, *TPK5*, and *KAT3* were up-regulated in the roots. These results suggest that distinct channel proteins and transporters were employed in the roots and leaves of common vetch to regulate ion homeostasis under salt stress. AKTs serve as the main potassium transporter responsible for facilitating the uptake of K^+^ by plant root cells from the outside [66]. *OsAKT1* maintains K^+^ balance in the plant by affecting the absorption of Na^+^ in roots, while *AtAKT2* functions to facilitate K^+^ loading and unloading in phloem tissues [67,68]. The K^+^ transporter (KT)/high-affinity K^+^ transporter (HAK)/K^+^ uptake protein (KUP) family is the major K^+^ acquisition system [69]. *OsHAK16* mediates K^+^ uptake and root-to-shoot translocation in a broad range of external K^+^ concentrations, thereby contributing to the maintenance of K^+^ homeostasis and salt tolerance in the rice shoots [70]. *OsHAK5* inhibits the uptake of Na^+^ but enhances the transportation of K^+^, whereas *AtHAK5* functions to transport K^+^ in the presence of high Na^+^ [69,71]. The Na^+^/H^+^ antiporter NHX is primarily located on the tonoplast to transport Na^+^ into the vacuole to avoid the accumulation of Na^+^ in the cytosol. Overexpression of *AtNHX1* leads to enhanced salt tolerance in transgenic plants [72]. Higher expression levels of *HKT1*, *AKT1*, *AKT2*, *HAK17*, *HAK25*, *NHX2*, *NHX6*, and *SKOR* in the leaves and those of *AKT2*, *HAK17*, *NHX7*, *HAK5*, and *TPK5* in the roots were observed in 460 compared to 429 after salt treatment, suggesting that the difference in gene expression levels in response to salinity was associated with the differential salt tolerance between 460 and 429. 

Salinity triggers the rapid production of ROS in plants. A low level of ROS usually acts as an essential signal in response to salt stress, but excessive accumulation of ROS imposes oxidative damage to proteins, lipids, and nucleic acids, causing cell death [73]. The antioxidant defense system plays a crucial role in the maintenance of ROS homeostasis under stress conditions. The major antioxidant enzymes include SOD, CAT, APX, and GST [74,75]. Higher levels of SOD, CAT, and APX and lower ROS accumulation were observed in 460 than in 429 under salt conditions [25]. Higher expression levels of genes encoding antioxidant enzymes such as *CAT3*, *SODCC*, and *APX4* were observed in 460 than in 429 in the present study, which is similar to results in potatoes [76]. These results in combination with previous investigations suggest that a higher antioxidant defense system induced by salinity was associated with salt tolerance in common vetch. 

## 5. Conclusions

The transcriptomic analysis provided insights into the molecular responses to salinity in common vetch by comparing a salt-tolerant line (460) and a salt-sensitive line (429). The common responses in common vetch and the specific responses associated with salt tolerance in 460 were analyzed. Processes such as plant hormone and MAPK signaling, phenylpropane biosynthesis, and galactose metabolism were identified as being commonly enriched KEGG pathways in response to salt stress. Moreover, several DEGs displayed unique expression patterns between 460 and 429, suggesting that they were associated with the differential salt tolerance between the two lines. Pathways involved in α-linolenic acid metabolism, carotenoid biosynthesis, the photosynthesis-antenna pathway, and starch and sucrose metabolism were specifically enriched in 460, and higher levels of accumulated soluble sugars in the leaves and lignin in the roots were observed in 460 than in 429 in response to salt stress. It is suggested that the above DEGs are associated with a higher salt tolerance in 460 than in 429.

## Data Availability

Data are contained within the article and its Supplementary Materials.

## Supplementary Materials

The following supporting information can be downloaded at https://www.mdpi.com/article/10.3390/plants13050714/s1.
