# Supplementary material for: Transcriptome Profiling Reveals Molecular Responses to Salt Stress in Common Vetch (Vicia sativa L.)
Source: Plants (Basel). 2024 Mar 3;13(5):714. doi: 10.3390/plants13050714 (PMC10935011; doi:10.3390/plants13050714)
Supplement: Supplementary file 1 [file plants-13-00714-s001.zip › plants-2853537-supplementary.pdf]

Supporting information

Table S1. Primers used for qRT-PCR analysis.

Gene name	Primer name	Sequence
CCR1	Y9361, forrward	AAGGAGCGAAGGAAAGACTAAC
	Y9362, reverse	TGCTAATGCCACATCCCTAAC
CCR2	Y9363, forrward	GTTAGAGGAACCCTCAGAAACC
	Y9364, reverse	GTTATGGGAAGACAGTGGCA
CAD1	Y9365, forrward	CTTGGGAGTGACGACGTTTAT
	Y9366, reverse	GATACGCCACTTCCATCGTAG
CAD2	Y9367, forrward	AGGTTCTAGAGGTGGGATCAA
	Y9368, reverse	TGATACTGTTCCTGTTGGTCAC
AKT2	Y9261, forrward	CATCATCCTACATCATCTTCATCTTTC
	Y9262, reverse	AGTTGCTTTCATGCATTCTTCTC
HAK17	Y9263, forrward	GTCTGCAATGGATGGAGTTAGA
	Y9264, reverse	TTATCTCCGCACTACATTGTCC
APX4	Y9351, forrward	GTCCTTGTAGTAAGGCAAGAAAGA
	Y9352, reverse	TATCACGGAGATGCGACTCA
CAT1	Y9353, forrward	CCCAAGTGGAAGTTCGGTATT
	Y9354, reverse	AGTAGTTGACGGTGCCTTTG
P5CS1	Y9355, forrward	GCGTTGGAAGCTGGATATGA
	Y9356, reverse	CTTATCGGGCTTGTCACTTCA
Unigene68614	Y7554, forrward	GCTAAAGCATTGAACAACAAAAGA
	Y7555, reverse	GCAAAGTTTGTCCCTTCACC

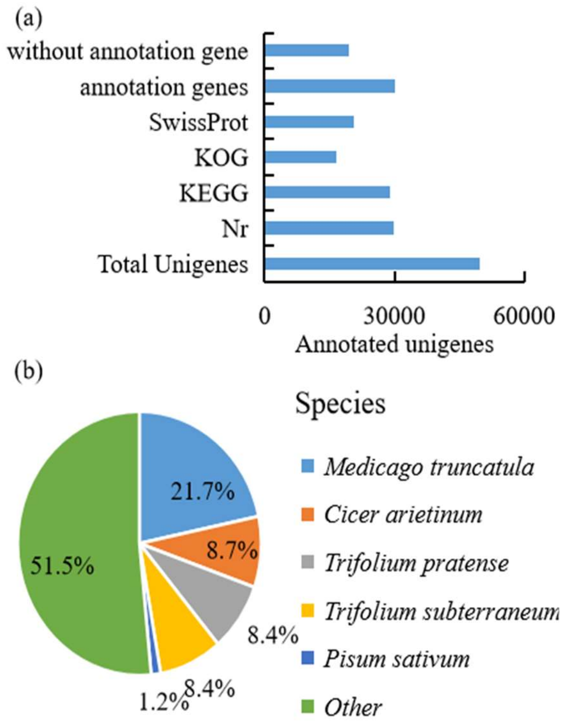

Figure S1. Annotation statistics and species distribution of all unigenes

(a) Statistics of unigenes annotation. (b) The species distribution of the total homologous sequences.

Figure S2

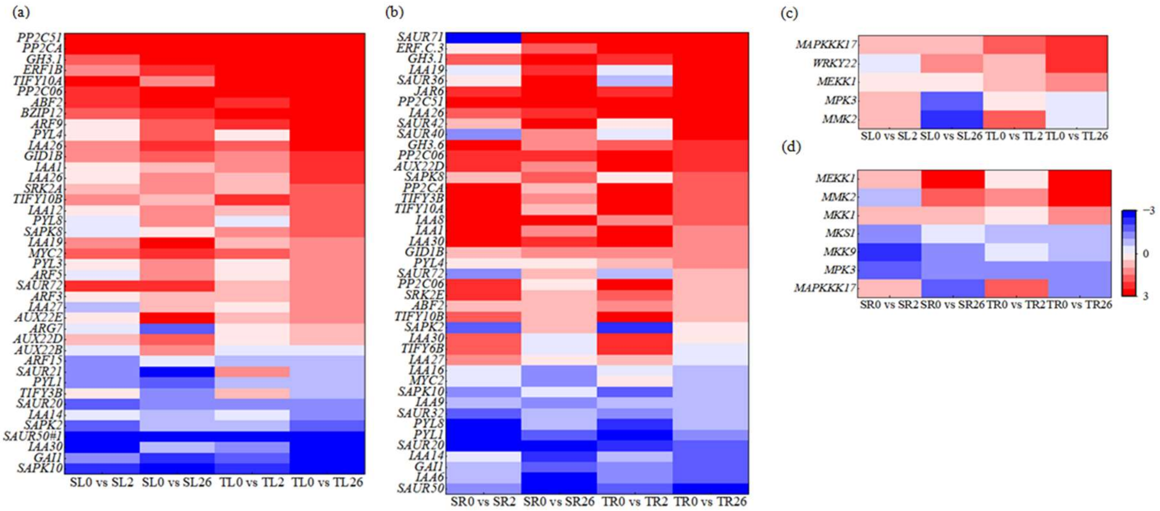

Figure S2. Heatmap of the DEGs involved in plant hormone and MAPK signaling pathways in two common vetch collections in response to salt stress.

The DEGs involved in plant hormone signal transduction in leaves (a) and roots (b) and MAPK signaling in leaves (c) and roots (d) were presented respectively. The color scale represents $\log_2$-transformed FPKM (fragments per kilobyte per million reads) values. The gradual change of the color indicates the different expression levels of DEGs, Red color indicates up-regulation, and blue color indicates down-regulation. SL, leaves in salt-sensitive collection 429; TL, leaves in salt-tolerant collection 460; SR, roots in salt-sensitive collection 429; TR, roots salt-tolerant collection 460. 0, 1, 2 represent 0, 2, 26 h after salt treatment, respectively.

Figure S3

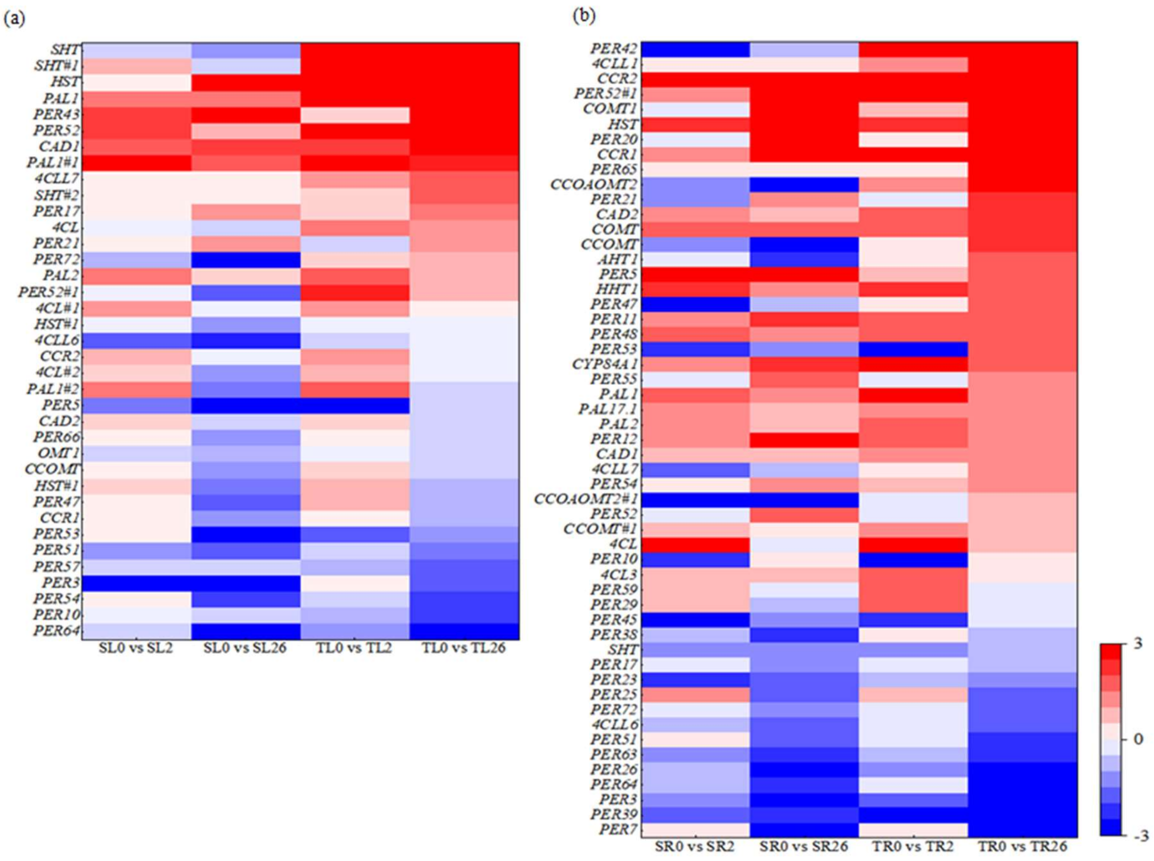

Figure S3. Heatmap of the DEGs involved in phenylpropanoid biosynthesis in two common vetch collections in response to salt stress

The DEGs involved in phenylpropanoid biosynthesis in leaves (a) and roots (b) were presented. The color scale represents log_2-transformed FPKM (fragments per kilobyte per million reads) values. The gradual change of the color indicates the different expression levels of DEGs. Red color indicates up-regulation, and blue color indicates down-regulation. SL, leaves in salt-sensitive collection 429; TL, leaves in salt-tolerant collection 460; SR, roots in salt-sensitive collection 429; TR, roots salt-tolerant collection 460. 0, 1, 2 represent 0, 2, 26 h after salt treatment, respectively.

Figure S4

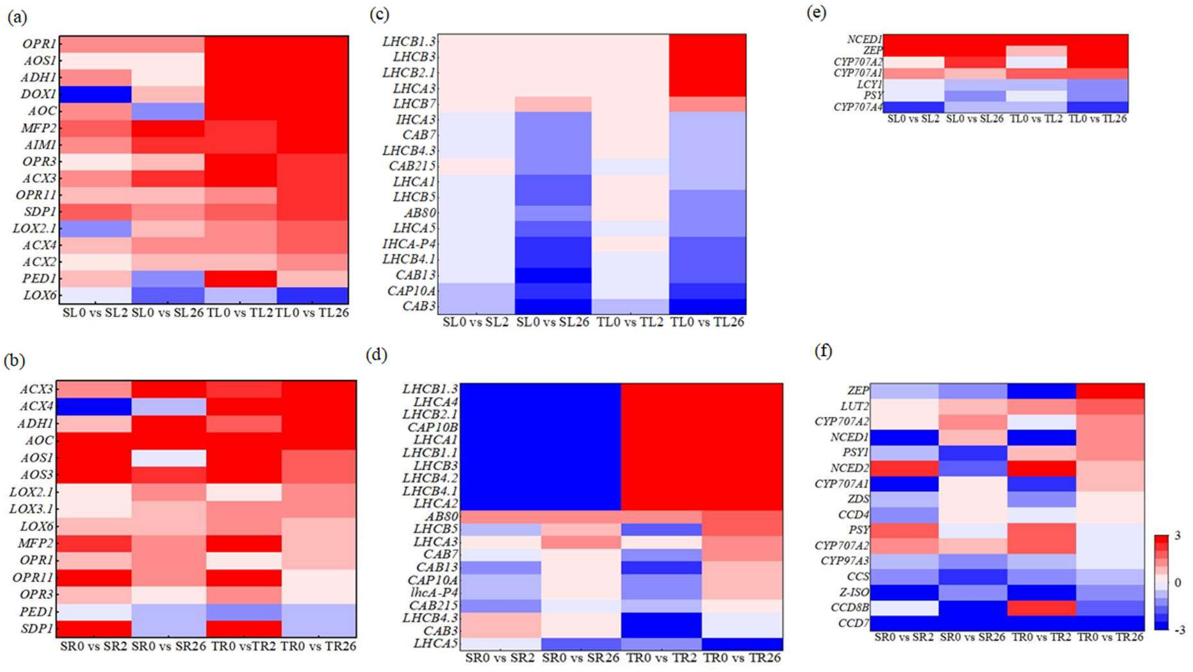

Figure S4. Heatmap of DEGs involved in alpha-linolenic acid metabolism, photosynthesis-antennain, and carotenoid biosynthesis of 429 and 460 in response to salt stress

The DEGs involved in alpha-linolenic acid metabolism (a, b), photosynthesis-antennain (c, d), and carotenoid biosynthesis (e, f) were presented. The data from leaves were represented in (a), (c), and (e), while those from roots in (b), (d), and (f). The color scale represents log_2-transformed FPKM (fragments per kilobyte per million reads) values. The gradual change of the color indicates the different expression levels of DEGs. Red color indicates up-regulation, and blue color indicates down-regulation. SL, leaves in salt-sensitive collection 429; TL, leaves in salt-tolerant collection 460; SR, roots in salt-sensitive collection 429; TR, roots salt-tolerant collection 460. 0, 1, 2 represent 0, 2, 26 h after salt treatment, respectively.
